# Supplementary material for: Complexome profiling of the Chlamydomonas psb28 mutant reveals TEF5 as an early PSII assembly factor
Source: Plant Cell. 2025 Mar 17;37(6):koaf055. doi: 10.1093/plcell/koaf055 (PMC12164590; doi:10.1093/plcell/koaf055)
Supplement: koaf055_Supplementary_Data [file koaf055_supplementary_data.zip › psb28_interactive.zip!/plots/intensity/qualityPlots/peptides/PSB27_Cre05.g243800.t1.2.html]

### Cre05.g243800.t1.2

Trivial name: PSB27  
SeqCoverage [median]: 22.50  
Mass: 19.09 kDa  
  
 

| Sequence | MissedCleavage | Mass [Da] | wtIntensity | muIntensity | Score |
| --- | --- | --- | --- | --- | --- |
| DINNWVAK | 0 | 958.49 | 14742.66 | 8621.99 | 180.93 |
| ELDDATLLLTR | 0 | 1258.68 | 35454.05 | 46206.89 | 213.80 |
| KDINNWVAK | 1 | 1086.58 | 1450.03 | 0.00 | 79.15 |
| LQKELDDATLLLTR | 1 | 1627.91 | 45638.55 | 109547.21 | 174.47 |
| VTLALDKDDPAK | 1 | 1284.69 | 0.00 | 1565.12 | 47.29 |
| VTLALDKDDPAKEDSVK | 2 | 1842.96 | 45693.89 | 67217.44 | 174.68 |
|  |  |  | 142979.2 | 233158.6 |  |
